# Well prepared for work? Junior doctors' self-assessment after medical education

**DOI:** 10.1186/1472-6920-11-99

**Published:** 2011-11-24

**Authors:** Elke B Ochsmann, Ulrike Zier, Hans Drexler, Klaus Schmid

**Affiliations:** 1 Institute of Occupational and Social Medicine, Medical Faculty, RWTH Aachen University, Pauwelsstrasse 30, D-52074 Aachen, Germany; 2 Institute of Occupational, Environmental and Social Medicine, Mainz University Medicine, Obere Zahlbacher Strasse 67, D-55131 Mainz, Germany; 3 Institute of Occupational, Environmental and Social Medicine, University of Erlangen-Nuremberg, Schillerstrasse 25/29, D-91054 Erlangen, Germany

## Abstract

**Background:**

Apart from objective exam results, the overall feeling of preparedness is important for a successful transition process from being a student to becoming a qualified doctor. This study examines the association between self-assessed deficits in medical skills and knowledge and the feeling of preparedness of junior doctors in order to determine which aspects of medical education need to be addressed in more detail in order to improve the quality of this transition phase and in order to increase patient safety.

**Methods:**

A cohort of 637 doctors with up to two years of clinical work experience was included in this analysis and was asked about the overall feeling of preparedness and self-assessed deficits with regard to clinical knowledge and skills. Three logistic regression models were used to identify medical skills which predict the feeling of preparedness.

**Results:**

All in all, about 60% of the participating doctors felt poorly prepared for post-graduate training. Self-assessed deficits in ECG interpretation (aOR: 4.39; 95% CI: 2.012-9.578), treatment and therapy planning (aOR: 3.42; 95% CI: 1.366-8.555), and intubation (aOR: 2.10; 95% CI: 1.092-4.049) were found to be independently associated with the overall feeling of preparedness in the final regression model.

**Conclusions:**

Many junior doctors in Germany felt inadequately prepared for being a doctor. With regard to the contents of medical curricula, our results show that more emphasis on ECG-interpretation, treatment and therapy planning and intubation is required to improve the feeling of preparedness of medical graduates.

## Background

The successful completion of a medical school education should provide students with a level of knowledge and skills necessary to fulfil a junior doctor's daily duties at hospital. While the level of training is usually evaluated in medical exams, it stands to reason that the results of these exams do not represent the whole truth of how well a medical student feels prepared for doing a doctor's job. In fact, different researchers demonstrated that exam results do not correlate with a resident's level of confidence or feeling of preparedness [e. g. [1,2]]. But apart from a successful graduation, a positive self-assessment of one's abilities is especially important, as it is likely to influence career choice, performance, and persistence in areas where the incompetencies are perceived [3,4]. This is the reason why - besides of passing the final exams - junior doctors should also feel well-prepared for their job and experience as few deficits as possible in their everyday tasks.

In 2003, Jungbauer et al. [5] reported that only about one third of the graduates of seven German universities felt well or very well prepared for being a doctor after finishing medical school, and many graduates criticized the lack of practical relevance of the curriculum with regard to their upcoming career. This step from being a student to becoming a junior doctor is often perceived as problematic [6], and was argued to be responsible for psychosocial distress especially in the first year after graduation [7-10]. Therefore, further research should generally be directed at the transition period from being a medical student to being a medical doctor and especially at identifying factors which are beneficial for becoming a junior doctor in practice.

Until now, it is unclear if a correlation between preparedness and self-assessed deficits in core competencies of medical education exists. Therefore, this evaluation focuses on the hypothesis that specific areas of medical care (core competencies) can be identified, which influence the overall feeling of preparedness, and might therefore also influence the mental health of junior doctors and the quality of patient care provided by these doctors [11]. The examination of these factors might give new insights into the underlying aspects of "feeling prepared for being a doctor".

## Methods

### Junior doctors

In September 2006, we mailed self-administered questionnaires to 1,494 doctors, who were registered in Bavaria for the first time in 2005. Names and addresses of the doctors were obtained from the Bavarian Medical Board Register (Bayerische Landesärztekammer). In order to participate, doctors had to send back the questionnaire anonymously in a pre-paid envelope. Neither the questionnaire nor the envelope allowed backtracking. As we intended to interpret exclusively the data of young doctors with one to a maximum of two years of postgraduate clinical experience, we selected the returning answers of those who had finished their medical education with the state medical examination in spring or autumn 2005. For the analysis presented in this paper, we furthermore excluded participants who did not work in patient care.

### Ethics

By anonymously sending back the filled-in questionnaire, participating junior doctors provided informed consent. Ethical clearance for this anonymous survey of junior doctors was received from the Ethical Committee, Medical Faculty, RWTH Aachen University.

### Preparedness (outcome variable)

The feeling of preparedness after finishing medical education was used as outcome variable for this analysis. It was surveyed with a dichotomous one-item question: "After finishing medical school, did you feel well prepared for being a doctor?" (yes/no).

### Self-assessed deficits in clinical competencies (predictors)

In order to collect data on self-assessed deficits according to relevant contents of medical education (core competencies), one senior physician, one resident physician, and one student developed a catalogue of 15 items of skills/knowledge to be acquired at medical school. The respective items were selected according to a proposal of German Medical students on the contents of a core curriculum of medical education [12]. The items were reviewed for completeness and sufficiency by ten junior doctors working at the Medical Faculty of Erlangen University. The following items were integrated in the questionnaire (alphabetical order):

▪ differential diagnosis: how to check possible symptom-related underlying medical diagnoses

▪ documentation & quality control: how to document medical results and how to use standards and procedures to ensure satisfactory performance

▪ ECG interpretation: how to systematically interpret a 12-lead ECG

▪ hygiene: how to manage hospital hygiene and infection control

▪ intubation: how and when to use an intubation set and airway management

▪ laboratory analysis: how to draw samples and how to interpret laboratory tests

▪ medical counselling to individual questions: how to gather and pass on information with regard to patient lifestyle, background, environment

▪ medical history taking: how to take a complete and systematic medical history

▪ patient management/difficult communication: how to communicate clearly, sensitively, and effectively with patients and relatives and how to break bad news

▪ pharmacotherapy: how to treat diseases through the administration of drugs, how to calculate drug dosages

▪ physical examination: how to perform a full physical examination

▪ (cardiopulmonary) resuscitation: how to react in case of an emergency, how to perform resuscitation

▪ social medicine & rehabilitation: how to use the health care system, how to plan rehabilitation contents, how to select rehabilitation institutions

▪ therapy or treatment planning: how to make clinical decisions based on symptoms, how to create a step-by-step treatment plan and how to manage the patient from admission to discharge

▪ X-ray interpretation: how to interpret X-rays, CT scans and MRTs

All of the selected 15 items were listed below the statement "At the beginning of my career I experienced deficits in..." and could be answered on a 6-point scale: "always", "often", "sometimes", "seldom", "never" or "not relevant". For further analyses, all predictors were dichotomized. The answers "always", "often", and "sometimes" were merged and coded as "deficits", the answers "seldom" or "never" were merged and coded as "no deficits". The answers "not relevant" were denoted, but excluded from multivariate analysis.

### Personal and work-related factors (confounders)

The following personal factors were considered as possible confounders for this evaluation: age (< 30 years, ≥ 30 years), gender, personal living conditions (living with a partner vs. living without a partner), and children under the age of 15 living in the household (yes vs. no). Apart from that, selected workplace-related variables were also regarded as confounders, as they might influence the feeling of preparedness, as well as self-assessed deficits. For example, it might be more important to know basic resuscitation in hospitals with few colleagues (primary care hospital) when compared to university hospitals, or it might be more important to have knowledge about ECG interpretation in internal medicine when compared to psychiatry. Therefore, the following work-related factors were included as confounders: occupational setting (university hospital, tertiary care hospital, secondary care hospital, primary care hospital, private practice; used as dummy variable), specialty (internal medicine, general surgery, trauma/orthopaedic surgery, anaesthesiology, gynecology/obstetrics, pediatrics, neurology, psychiatry, other specialties; used as dummy variable), number of student internships (four (required for all students), five, six or more), previous or additional vocational training in the medical field (none, student assistant/night watch, vocational education (e. g. as a nurse); used as dummy variable).

Because of the retrospective assessment of the feeling of preparedness, we also included the following workplace factors in the regression analyses, as these factors might have led to retrospective bias of the results: frequency of in-house training (at least once per month, less than once per month), performance-related feedback of superiors (never, seldom/sometimes, often/always; used as dummy variable), support by colleagues and superiors (no/little support, enough/a lot of support).

### Statistical methods

Data was analysed using SPSS version 18.0. Descriptive statistics and chi-square tests were calculated to identify interactions between predictors, confounders and the outcome variable. P values < 0.05 were considered statistically significant. Three multivariate logistic regression models (backwards selection) were used to identify independent associations between self-assessed deficits (predictors), personal and work-related variables (confounders) and "feeling well prepared" (outcome variable) in all participants. In the first model, only the predictors (self-perceived deficits) were entered in the multivariate model. In the second model, all predictors and all personal confounders were entered in the model. In the final third model, all predictors and all personal and work-related confounders were entered in the model. Adjusted Odds Ratios and according 95% confidence intervals were calculated as risk estimates (aOR; 95% CI).

## Results

A total of 792 doctors participated in this survey (response rate: 53%), 637 of whom had been working in patient care for less than two years, and were therefore included in this analysis of clinically working junior doctors. The age of the participants ranged between 26 and 50 years ((mean ± standard deviation): 29.3 ± 2.2 years); 276 of the participants were male (43.8%) (table 1).

**Table 1:** Association between personal factors (confounders) and preparedness after medical education (descriptive analysis)

		Did you feel well prepared after finishing medical education?				
		yes	%*	no	%*	Chi^2^
gender	female	113	32.3	237	67.7	0.222
	male	102	37.0	174	63.0	

age	< 30 years	168	36.7	290	63.3	0.038
	≥ 30 years	47	27.8	122	72.2	

living situation	with partner	125	34.2	240	65.8	0.944
	without partner	88	34.0	171	66.0	

children < 15 years in household	yes	25	36.2	44	63.8	0.705
	no	186	33.9	362	66.1	

* row percentage

### Feeling of preparedness after medical education

Altogether 34.2% (n = 215) of the participants reported that they felt well prepared for their up-coming medical career at the end of medical school, whereas 65.8% (n = 413) of them felt inadequately prepared. With regard to the selected personal and work-related factors, junior doctors who were older were more likely to feel inadequately prepared (p = 0.038) (table 1). Furthermore, the feeling of preparedness was associated with the frequency of performance-related feedback by superiors (p = 0.002), as well as the amount of support from colleagues and superiors (p = 0.001). More performance-related feedback and more support were both related to a better feeling of preparedness. All other confounders showed no significant association with the feeling of preparedness (table 2).

**Table 2:** Association between workplace factors and inter-personal factors at the workplace (confounders) and preparedness after medical education (descriptive analysis)

		Did you feel well prepared after finishing medical education?				
		yes	%*	no	%*	Chi^2^
occupational setting	university	73	42.4	99	57.6	0.092
	tertiary care hospital	49	31.8	105	68.2	
	secondary care hospital	46	32.9	94	67.1	
	primary care hospital	40	28.2	102	71.8	
	practice	7	35.0	13	65.0	

specialty	internal medicine	52	28.0	134	72.0	0.238
	general surgery	40	39.6	61	60.4	
	trauma surgery/orthopedic surgery	14	32.6	29	67.4	
	anesthesiology	27	35.5	49	64.5	
	gynecolocy/obstetrics	18	36.0	32	64.0	
	pediatrics	15	44.1	19	55.9	
	neurology	14	45.2	17	54.8	
	psychiatry	6	20.7	23	79.3	
	other specialties	26	34.1	45	65.9	

no. of internships	4 internships (required)	119	34.8	223	65.2	0.204
	5 internships	60	30.0	140	70.0	
	≥6 internships	34	42.5	46	57.5	

(previous) medical experience	none	44	27.3	117	72.7	0.153
	student assistant	66	36.5	115	63.5	
	several jobs as student assistant	68	35.8	122	64.2	
	vocational education (nurse etc)	37	39.8	56	60.2	

frequency of in-house training	at least once per month	140	37.1	237	62.9	0.061
	less than once per month	75	29.9	176	70.1	

performance-related feedback by superiors	never	31	22.6	106	77.4	0.002
	seldom or sometimes	155	36.0	275	64.0	
	often or always	28	46.7	32	53.3	

support by supervisors and colleagues	enough or a lot of support	187	37.3	314	62.7	0.001
	little or no support	28	22.0	99	78.0	

* row percentage

### Self-assessed deficiencies

**Figure 1:**
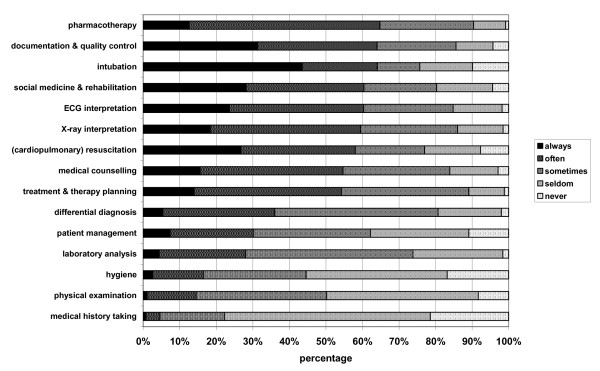
**Answers of junior doctors (up to two years postgraduate training) when asked about self-assessed deficits according to clinical knowledge and skills (sorted from the item where most junior doctors reported deficiencies "always" and "often" (pharmacotherapy) to the item where the least junior doctors reported deficiencies "always" and "often" (medical history taking))**.

The five most frequently reported items in which junior doctors "always" experienced deficiencies were: "intubation" (43.5%), "documentation & quality control" (31.3%), "social medicine & rehabilitation" (28.2%), "resuscitation" (26.8%), and "ECG interpretation" (23.6%) (Figure 1). More than one fifth of the participating junior doctors "always" experienced deficits in these areas. Most self-assessed deficits showed a statistically significant association with the feeling of preparedness in the χ^2^-test, with the exception of "social medicine & rehabilitation" (p = 0.353), and "hygiene" (p = 0.533) (table 3).

**Table 3:** Association between self-assessed deficits in clinical knowledge or skills (predictors) and preparedness after medical education (descriptive analysis)

		Did you feel well prepared after finishing medical education?					
		**yes**	(%)*	**no**	(%)*	total number (%)**	**Chi^2^**

I experience deficits in ...							

**differential diagnosis**	no	61	49.6	62	50.4	123 (19.6%)	<0.001

*irrelevant (n = 2 (.3%))*	yes	153	30.4	350	69.6	503 (80.4%)	

**documentation & quality control**	no	37	44.0	47	56.0	84 (14.5%)	0.048

*irrelevant (n = 50 (7.8%))*	yes	163	32.9	332	67.1	495 (85.5%)	

**ECG interpretation**	no	53	58.2	38	41.8	91 (15.3%)	<0.001

*irrelevant (n = 32 (5.0%))*	yes	151	29.9	354	70.1	505 (84.7%)	

**hygiene**	no	120	35.5	218	64.5	338 (55.4%)	0.533

*irrelevant (n = 18 (2.8%))*	yes	90	33.1	182	66.9	272 (44.6%)	

**intubation**	no	57	49.6	58	50.4	115 (24.8%)	<0.001
*irrelevant (n = 164 (25.7%))*	yes	95	27.2	254	72.8	349 (75.62%)	

**laboratory analysis**	no	69	43.7	89	56.3	158 (25.7%)	0.003
*irrelevant (n = 12 (1.9%))*	yes	141	30.9	316	69.1	457 (74.3%)	

**medical counselling**	no	36	43.4	47	56.6	83 (16.4%)	0.048
*irrelevant (n = 123 (19.3%))*	yes	136	32.2	287	67.8	423 (83.6%)	

**medical history taking**	no	186	35.5	297	61.5	483 (77.8%)	<0.001
*irrelevant (n = 7 (1.1%))*	yes	26	18.8	112	81.2	138 (22.2%)	

**patient management**	no	102	43.0	135	57.0	237 (38.1%)	<0.001
*irrelevant (n = 6 (.9%))*	yes	111	28.8	274	71.2	385 (61.9%)	

**pharmacotherapy**	no	30	49.2	31	50.8	61 (9.8%)	0.009
*irrelevant (n = 4 (.6%))*	yes	183	32.5	380	67.5	563 (90.2%)	

**physical examination**	no	132	42.6	178	57.4	310 (50.2%)	<0.001
*irrelevant (n = 10 (1.6%))*	yes	80	26.0	228	74.0	308 (49.8%)	

**(cardiopulmonary) resuscitation**	no	62	51.2	529	48.8	121 (23.2%)	<0.001
*irrelevant (n = 107 (16.8%))*	yes	118	29.5	282	70.5	400 (76.8%)	

**social medicine & rehabilitation**	no	41	38.7	65	61.3	106 (19.9%)	0.353
*irrelevant (n = 94 (14.8%))*	yes	145	33.9	283	66.1	428 (80.1%)	

**treatment & therapy planning**	no	41	62.1	25	37.9	66 (11.0%)	<0.001
*irrelevant (n = 29 (4.6%))*	yes	162	30.4	371	69.6	533 (89.0%)	

**x-ray interpretation**	no	43	50.6	42	49.4	85 (14.0%)	0.001
*irrelevant (n = 22 (3.5%))*	yes	167	32.1	354	67.9	521 (86.0%)	

* row percentage; ** column percentage

### The association between self-assessed deficits and preparedness

Regression model 1 (table 4) examined the association between preparedness and self-assessed deficits only, and showed statistically significant results for deficits in ECG-interpretation (aOR: 2.73; 95% CI: 1.34-5.57), deficits in treatment and therapy planning (aOR: 3.93; 95% CI: 1.75-8.82) and deficits in intubation (aOR: 2.42; 95% CI: 1.33-4.42). Regression model 2 further included personal factors as confounders and found that, apart from the above mentioned self-assessed deficits, which remained in the model, living without a partner was associated with feeling unprepared (aOR: 1.98; 95% CI: 1.12-3.50). The third and final regression model additionally included all structural and interpersonal workplace factors (see also table 3) and found the following factors to be independently associated with the postgraduate feeling of preparedness: self-perceived deficits in ECG-interpretation (aOR: 4.39, 95% CI: 2.01-9.58), deficits in treatment and therapy planning (aOR: 3.42, 95%CI: 1.37-8.56), deficits in intubation (aOR: 2.10, 95% CI: 1.09-4.05), living without a partner (aOR: 1.86, 95% CI: 1.03-3.35), performance related feedback by superiors ((seldom/sometimes vs. never) aOR: 0.49, 95% CI: 0.25-0.96) and the number of internships ((more than six internships vs. four) aOR: 0.40; 95% CI: 0.18-0.86) (table 4). All of these factors were retained in the backwards selection as independent predictors.

**Table 4:** Logistic regression models with backwards selection for predicting the influence of self-assessed deficits in skills or knowledge on preparedness for working as a medical doctor (adjusted Odd's ratios; 95% confidence intervals)*

			Model 1*		Model 2*		Model 3*	
			
			"Did you feel well prepared after finishing medical education (yes/no)?" Predictors: self-perceived deficiencies in clinical skills or knowledge (table 3)		"Did you feel well prepared after finishing medical education (yes/no)?" Predictors: self-perceived deficiencies in clinical skills or knowledge (table 3) Confounder: age, gender, living situation, children living in household (table 1)		"Did you feel well prepared after finishing medical education (yes/no)?" Predictors: self-perceived deficiencies in clinical skills or knowledge (table 3) Confounder: Model 2 + workplace factors (table 1 + table 2)	
			
			aOR	95%CI	aOR	95%CI	aOR	95%CI
deficits in...	ECG interpretation	no	1.00		1.00		1.00	
		yes	2.73	1.335-5.569	3.01	1.428-6.345	4.39	2.012-9.578
	
	treatment & therapy planning	no	1.00		1.00		1.00	
		yes	3.93	1.749-8.815	4.14	1.775-9.673	3.42	1.366-8.555
	
	physical examination	no	1.00		1.00			
		yes	1.66	0.992-2.768	1.66	0.979-2.820		
	
	intubation	no	1.00		1.00		1.00	
		yes	2.42	1.330-4.419	2.46	1.317-4.584	2.10	1.092-4.049

personal factors	living situation	with partner			1.00		1.00	
		without partner			1.98	1.124-3.502	1.86	1.033-3.346

workplace factors	performance-related feedback by superiors	never					1.00	
		seldom, sometimes					0.49	0.249-0.961
		often always					0.52	0.191-1.438

	number of internships	four (required)					1.00	
		five					1.47	0.804-2.674
		six or more					0.40	0.181-0.860

* **model 1: **all factors of table 3 were included; **model 2: **all factors of table 1 and table 3 were included; **model 3: **all factors of tables 1 to 3 were included. The results depicted in table 4 are the factors which remain in the respective model after logistic regression with backwards selection.

## Discussion

As stated in Goldacre et al. [13], "most people starting a new professional job probably will, and probably should, feel unprepared to some extent". Nevertheless, this should not stop researchers and medical teachers from trying to provide the best preparation and education possible. This is especially important in the health care sector, where inexperience can lead to mistakes which affect patients' health [14,15]. In this study we analysed junior doctors' feeling of preparedness in relation to self-assessed deficits at the beginning of their clinical career. Preparedness and self-assessed deficits need to be addressed, as they can be associated with longer procedure time and higher costs, but, most of all, because they might induce more mental stress for junior doctors and might interfere with patient safety [14,15]. Junior doctors with at least some months of professional experience should be best qualified to retrospectively assess the ability of medical education to prepare them for being a doctor. Furthermore, they are more aware of deficits in certain areas, as they already had to answer the expectations of superiors, colleagues and patients and experienced the gap between medical school and clinical care [6]. The retrospective assessment of preparedness and deficits also represents a reflection on what has been experienced, rather than what is anticipated.

In the initial descriptive analysis of our participants, we found that approximately 66% of the participating junior doctors did not feel well prepared for their job after finishing medical education. This result supports the result of another German study by Jungbauer et al. [5] in which two thirds of the questioned alumni of seven medical universities in Germany reported to feel badly prepared for being a doctor, too. These high percentages were not supported by researchers from other countries [see for example [13,16]]. In comparison to our 2005 cohort of medical graduates, Goldacre et al. [13] found that only 23.8% of the 2005 cohort of UK medical school graduates (strongly) disagreed that their medical school had prepared them well for the jobs they had undertaken during the first postgraduate year. Goldacre et al. [13] also discovered differences in preparedness with regard to medical schools and differences with regard to the time of assessment. Cave et al. [17] found that 15% of respondents of their study felt poorly prepared by their medical school for starting work in the year 2005, whereas, in the same year, 61% of Irish interns felt insufficiently or poorly prepared [18]. The reasons for these differences in time and location are yet unclear. They might be associated with differences in medical education, with differences in expectations of students or junior doctors, with differences in questionnaire assessment, differences in health care systems, or they might also be due to response bias. Though the source of these differences is not clear yet, they have to be kept in mind when comparing results between different countries or different times of assessment.

We also found that many participants experienced deficits in important areas of clinical skills or knowledge (especially ECG interpretation, social medicine & rehabilitation, documentation & quality control, resuscitation, intubation). Interestingly, only one of the items, namely social medicine & rehabilitation, was not statistically associated with the feeling of preparedness in the χ^2^-test. Therefore, it seems as if German junior doctors, though they find themselves lacking an adequate amount of knowledge in social medicine & rehabilitation, do not perceive this area as essential for being well prepared for patient care. American students, too, reported deficits in knowledge of the U.S. health care system (knowledge of health care systems being a part of the subject "social medicine" in Germany) [19]. Altogether 96% of American students felt that understanding health policy is important and approximately 50% were dissatisfied with medical school course work. Nevertheless, this study did not assess the association between this dissatisfaction and the feeling of preparedness.

A study by Hastings et al. [20] found the generation of appropriate working diagnoses and the consideration of physical, social and psychological factors to be the two most frequent consultation weaknesses in students. In our study these weaknesses are, amongst others, represented in the topics "deficits in treatment or therapy planning", "deficits in differential diagnosis", and "deficits in social medicine and rehabilitation", three of the areas in which a large percentage of junior doctors reported deficits, respectively. With regard to resuscitation and intubation, our findings are also supported by an investigation of Hayes et al. [21] where 49% of internal medicine residents in Canada did not feel adequately trained to lead a cardiac arrest team. Similar to the results of Bojanić et al. [22] though, the participants of our study felt well prepared for history taking and physical examination. Because of the above outlined similarities, we think that these results are, at least to some extent, also true for other young doctors.

Our main hypothesis was that post-graduates' feeling of preparedness might be especially influenced by specific contents of the medical curriculum. Indeed, out of a list of 15 items of medical education, we found that especially self-assessed deficits in ECG interpretation, deficits in therapy planning, and deficits in intubation were independently associated with feeling poorly prepared for the job as a clinically working junior doctor, irrespective of confounders like age, gender, chosen specialty, or previous work experience in the medical field. These results are in accordance with findings of Hoppe et al. [16], who found significant positive correlations between satisfaction during the clinical part of medical education and the feeling of having appropriate skills regarding physical examination, acute critical situations, therapy planning, communication with colleagues and critical evaluation of information. An interesting finding was that deficits in intubation, rather than resuscitation in general, showed a significant influence on the feeling of preparedness in our study. Other authors, too, addressed emergency training in general as possible obstacle for the transition from medical student to practicing doctor and found that students valued acute emergency training in a preparation programme after graduation [23]. Taking into account that the range of experience might be due to the chosen specialty, it should be considered that emergency medicine or acute trauma were reported to be posts predominantly held by men, while women tend to choose "non-invasive" posts more often [24]. Nevertheless, deficits in intubation turned out to be a "gender-independent" factor in our multivariate analysis, and should therefore be considered more intensely in medical curricula or in practical settings. The other two items, ECG interpretation and therapy planning, are to be considered as core competencies of medical education. In the long term, the three items should be strengthened in medical curricula in Germany in order to improve junior doctors' feeling of preparedness after medical education.

Our study has some limitations which should be mentioned. The response rate was rather low, so it remains unclear if the participants of this study represent a certain subgroup of doctors who feel more or less prepared than the non-respondents (response bias). Furthermore, the anonymous approach did not allow for evaluation of the non-respondents. Nevertheless, the overall effective response rate can be regarded as adequate for survey studies [25] and corresponds to previous results of response rates of physician surveys. Another limitation is the reliability on self-reported ratings of deficits and preparedness by junior doctors. Thus, results may also be subject to bias in terms of response style and common method variance [26,27]. However, Shubert et al. found self-reported high levels of preparedness to be correlated with good performance [28], a finding which has to be regarded in the context of patient safety. Finally, the retrospective evaluation of preparedness, which we used in our study, may lead to bias. Therefore, we included personal and workplace factors as possible confounders into regression analyses, as these factors might have influenced the retrospective appraisal of preparedness after finishing medical education. Feedback of superiors, as well as living without a partner did indeed turn out to be associated with the feeling of preparedness, demonstrating the complex interactions which have to be considered when using self-assessments. Feedback was also identified as influencing factor on preparedness by other authors [29], stressing the importance and the benefit of the direct interaction between superiors and junior doctors, e. g. during "bedside teaching". Nevertheless, the inclusion of the above mentioned confounders did not alter the effect of deficits in the three identified core competencies on the feeling of preparedness, which underlines their independent influence.

## Conclusions

After finishing medical school, many German junior doctors do not feel well prepared for their career. Self-assessed deficits in intubation, ECG interpretation and therapy planning were found to be independently associated with a poor feeling of preparedness, and suggest that preparedness might be improved by addressing, stressing, and training these specific clinical competencies during medical education and beyond in order to alleviate the stress of the transition period between medical school and being a fully-fledged doctor, and in order to improve patient safety.

## Competing interests

The authors declare that they have no competing interests.

## Authors' contributions

HD, EBO, and KS designed and conducted the study, EBO and UZ conducted the statistical analysis. All four authors contributed to the writing of the paper and approved of the final manuscript.

## Pre-publication history

The pre-publication history for this paper can be accessed here:

http://www.biomedcentral.com/1472-6920/11/99/prepub
